# Prospective evaluation of a novel device for ultrasound-guided percutaneous treatment of carpal tunnel and trigger finger disease. Efficacy and safety of sono-instruments®

**DOI:** 10.1007/s40477-023-00851-y

**Published:** 2024-04-10

**Authors:** Fabian Moungondo, Hannah Van Rompaey, Mohamad K. Moussa, Frédéric Schuind

**Affiliations:** 1 Department of Orthopedics and Traumatology, Université Libre de Bruxelles, Erasme University Hospital, Brussels, Belgium; 2 Clinique du Sport, 36 boulevard saint marcel, 75005 Paris, France

**Keywords:** Percutaneous Treatment, Carpal Tunnel Syndrome, Trigger Finger, Ultrasound-guided Surgery, Sono-Instruments®, Minimally Invasive Surgery, Hand Surgery, Efficacy and Safety, Functional Recovery

## Abstract

**Purpose:**

To evaluate the safety and effectiveness of percutaneous release procedures under sonography using Sono-Instruments® in the treatment of carpal tunnel syndrome (CTS) and trigger finger (TF).

**Methods:**

Prospective study involving 30 patients, divided into two groups (15 CTS, and 15 TF). The primary outcomes were surgical performance-related outcomes (visibility, ease of use, satisfaction, duration) using Sono-Instruments® and patient-related outcomes (pain, activity limitations, time to return to work, functional scores). Secondary outcomes included complications. Patients were followed for two months post-operatively.

**Results:**

In the CTS group, the average age of the patients was 58.7 years. The percutaneous release of the transverse carpal ligament was effectively completed in all cases, with excellent device performance and no adverse events. At one week, all patients could wash their hands, 80% could perform activities of daily living, and 80% of those working had returned to their activities. At two months, all patients had resumed all activities. Pillar pain was still present in 53.3%. In the TF group, the patients had an average age of 57.9 years. The percutaneous release of the A1 annular pulley was successful in all cases, with excellent device performance and no adverse events. At one week, all patients could wash their hands, 93.3% could do all activities of daily living, and 75% of those working were back to their professional activities. At two months, all patients were back to all activities of daily living and work. The DASH score was significantly improved at two months, compared to preoperative, for both groups (*p* < 0.001).

**Conclusion:**

Percutaneous sono-guided release using Sono-Instruments® is safe and efficient, and associated with quick functional recovery.

**Level of evidence:**

II.

## Introduction

Carpal tunnel syndrome (CTS) and trigger finger (TF) are common conditions that necessitate surgical treatment when conservative approaches fail [1]. The classic open procedures carry risks of iatrogenic injuries, wound and scar issues. Minimally invasive procedures were developed to reduce these complications, [2–10] but did not prove effective in reducing the risk of iatrogenic nerve lesions due to visibility issues, particularly in cases involving anatomical variations of the median nerve and its branches [11].

Recent advances in high-frequency sonography have significantly enhanced the visualization of important soft-tissue anatomical structures such as the median nerve, its terminal branches, the transverse carpal ligament (TCL), the Berrettini medio-ulnar anastomosis, and intrinsic hand muscles [12]. Sonography allows localizing the pulsating ulnar artery, an important anatomical landmark, and its prolongation, the superficial carpal arch that is also at danger at the distal end of the TCL, close to the Berrettini anastomosis [13]. At the metacarpophalangeal (MCP) level, flexor tendons and their gliding are easily observed, and dynamic impairment of gliding is evident in most TF cases [14]. Sonography also discloses in TF the usually thickened A1 pulley, and the location of digital nerves [15]. These progresses in ultrasound imaging have opened the possibility of sonographic-guided surgical treatment of CTS and TF [2, 3, 5, 9, 16]. Most devices on the market are endoscopic surgical tools adapted to sonography, with a knife-like component used to perform an antegrade or a retrograde release [2–4, 8, 16]. These instruments require a small incision and dissection to create the plane of insertion of the relatively bulky cutting instrument [2, 3, 5, 9, 16]. Some devices are not adapted to thick TCL or A1 pulleys. Straight sectioning exposes to the risk of iatrogenic nerve injury [17–19]. Excessive force during sectioning can damage anatomical structures beyond the cut, such as the superficial palmar arch and the Berrettini medio-ulnar anastomosis in CTS surgery, or the FDS in TF surgery [13, 19, 20].

**Fig. 1:**
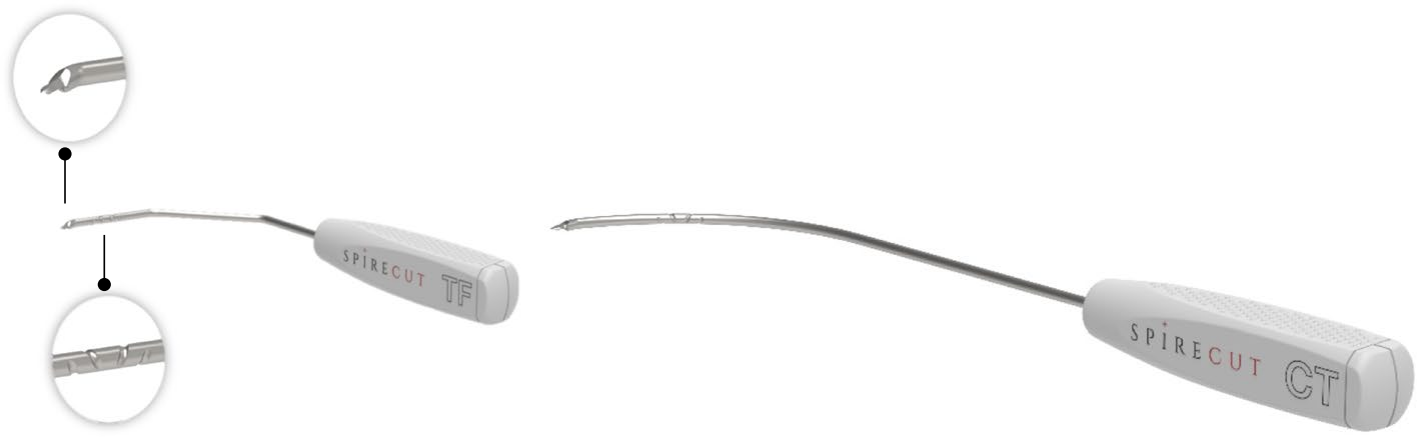
Sono-Instrument® (Spirecut AG): top, trigger finger model; bottom, carpal tunnel model. Note the shape of the extremity, allowing cutting in a plane of tensed ligamentous structures, and the flanges enhancing – like also the spiral groove – the visibility under sonography

The Sono-Instrument® (SI) (Spirecut AG, Muttenz, Switzerland) was designed to address these limitations. This innovative 1.5mm diameter medical device allows for truly percutaneous surgery, as all the operation is done through a simple puncture hole made by a 14G intravenous catheter puncture. There is no skin incision, there is no need for tissue dissection. There are two models, shaped and tailored, for CTS and TF procedures, respectively. The SI’s cutting extremity features a smooth flange, and its rod, a spiral groove, enhancing visibility under sonography. The device allows progressive sectioning of tensed fibers: the cutting extremity is not overly sharp, reducing the likelihood of injury to non-tight anatomical structures, such as the median nerve or a tendon, in case of inadvertent contact (Fig. 1).

**Fig. 2:**
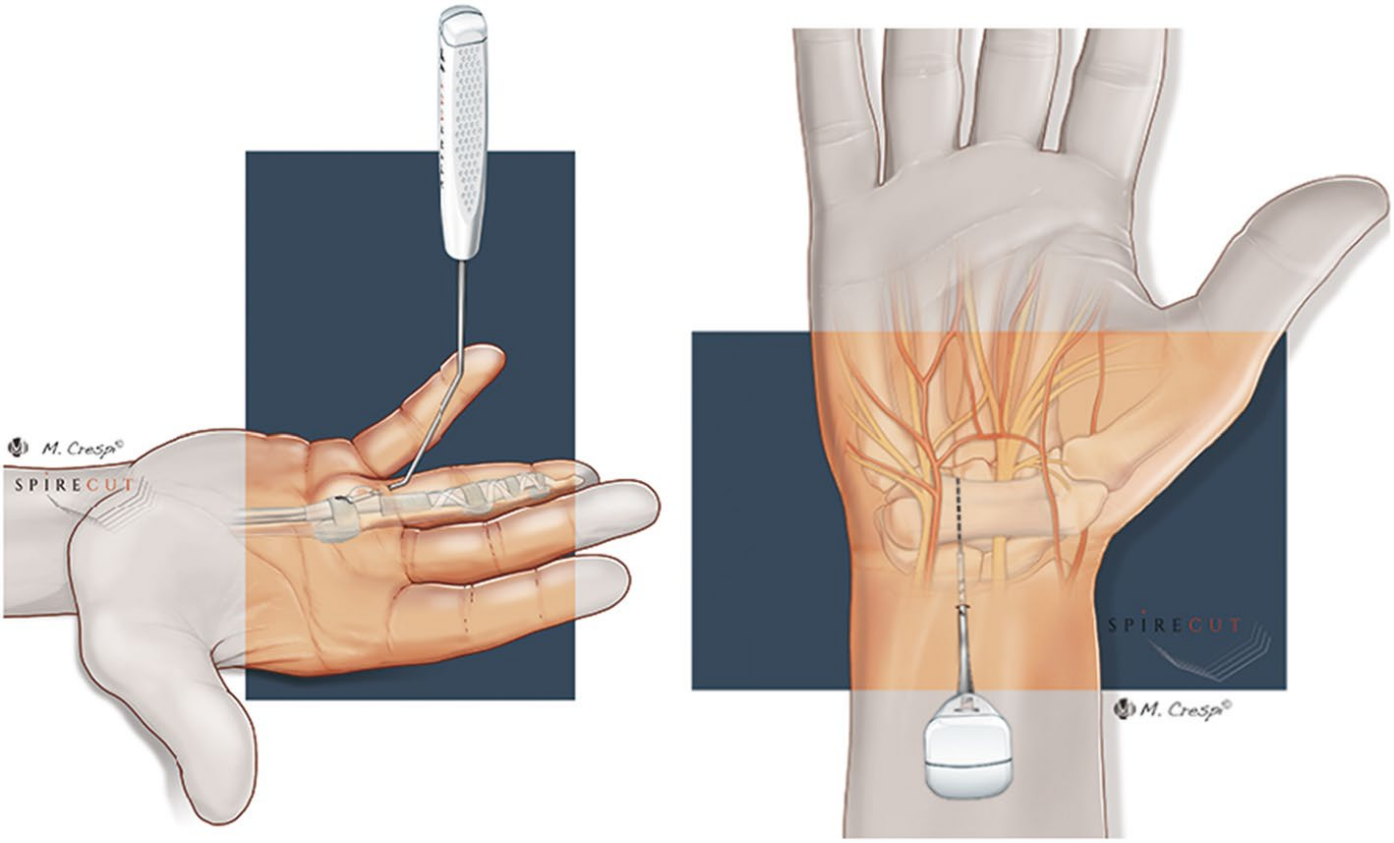
Surgical technique: left, TF A1 annular pulley release; right, CTS TCL release

For CTS, the SI allows for safe percutaneous antegrade release of the TCL while visualizing and protecting the superficial palmar arch, the Berrettini medio-ulnar anastomosis, and the thenar motor branch. The section of the TCL is done on its ulnar side, between the muscular insertions of the thenar and hypothenar muscles, far from the median nerve and limiting per-operative muscular bleeding. For TF surgery, the SI is introduced distally to the A1 pulley, at the MCP flexion crease for the digits, and at the mid proximal phalanx for the thumb. After having located the digital pedicles, the A1 retrograde section is safely performed (Fig. 2).

The purpose of this prospective study was to assess pre-CE-marking the safety and efficiency of the SI, in a series of 15 CTS and 15 TF patients.

## Materials and methods

### Study design

This pre-market, monocentric, prospective clinical investigation included 30 patients, 15 CTS and 15 TF. The inclusion and exclusion criteria are presented in Table 1.

**Table 1:** list of inclusion and exclusion criteria for the study

Inclusion criteria	
CTS	TF
Adults ≥ 18 years of age	Adults ≥ 18 years of age
Primary CTS confirmed by typical symptoms, signs and altered nerve conduction studies and pain and/or numbness in the hand which worsen at night (or are present only at night)	Typical signs and symptoms of TF caused by friction or blockade of flexor tendon(s) in digital sheath, without flexion contracture over 30° of the proximal interphalangeal (or interphalangeal for the thumb) joint, confirmed by altered flexor tendon(s) gliding and/or increased thickness of A1 digital pulley under sonography

Exclusion criteria
Comorbidities – related
Dwarfism or participants with small size hand/finger-thumb
Past or active infection
Known allergic reaction to metals
Coagulation problems, with significant risk of per/postoperative bleeding Contra-indication to local anesthesia
Surgical site-related
Previous fracture or dislocation in the operated area or any affection causing malalignment or distortion of the local skeleton due to trauma, arthritis, or other causes
Other known clinical risks outweighing the expected clinical benefits or increasing the risk of a postoperative lesion (e.g., tissue adhesions, anatomical abnormalities, neuro-vascular structures in the zone of the intended release, local tumor)
Previous attempt to treat the condition
Currently receiving treatment for CTS or TF
Disease-related
In case of TF: long evolution, with flexion contracture over 30° of the proximal interphalangeal (or interphalangeal for the thumb) joint
In case of CTS: severe median nerve dysfunction
Technique-related:
Insufficient sonographic identification of the operated tissue
Participant unable (vulnerable participant)/unwilling to provide informed consent
Participant enrolled in another study

The diagnosis of CTS was based on typical medical history and physical examination and confirmed by nerve conduction studies. The diagnosis of TF was based on characteristic clinical findings of finger catching and confirmed by pre-operative sonography, demonstrating thickened A1 pulley (> 0.62mm[21]) and/or impaired flexor tendon(s) gliding.

### Outcome measurements

The primary outcomes measurements of the study, assessed by an independent observer, were divided into two categories: surgical performance-related outcomes, and patient-related outcomes. Surgical performance-related outcomes included the visibility of the SI under sonography, the ease of positioning, maneuverability, and efficiency of performing the intervention, the satisfaction of the surgeon, and the duration of the procedure (duration from beginning of local anesthesia to the end of the surgical release). Patient-related outcomes included: (1) pain, assessed before and after the procedure (at 1 week and 2 months) using the Numeric Pain Rating Scale (NRS), and documentation of painkiller and anti-inflammatory use (at 1 week and 2 months); (2) activity limitations (at 1 week and 2 months); (3) time to return to work; (4) time to disappearance of dysesthesia for CTS patients, and to disappearance of triggering for TF patients (according to Quinnel’s classification [22] and Modified Patel and Bassini’s grading system [23]). Functional scores included the Disabilities of the Arm, Shoulder, and Hand (DASH) score [24], Boston Carpal Tunnel Questionnaire Symptom Severity (BCTQ-SS) [25], and Boston Carpal Tunnel Questionnaire Functional Scale (BCTQ-FS) [25].

Secondary outcome measurements included complications of the procedure such as peri-operative bleeding, nerve injury, stiffness, infection, symptomatic recurrences and re-interventions.

### Surgical technique

#### General consideration

All patients were operated on by the same surgeon (FM), experienced in sono-guided procedures, in a day clinic operative room, under local anesthesia, without tourniquet. No form of sedation was used, simply reassurance by constant explanations by the surgeon. The surgeon first performed a new sonography (as most CTS patients and all TF patients had had sonography before the operation day, in outpatient clinic), to identify important landmarks, structures at risk, anatomical variations and abnormalities.

#### CTS surgery: (Figs. 3, 4)

**Fig. 3:**
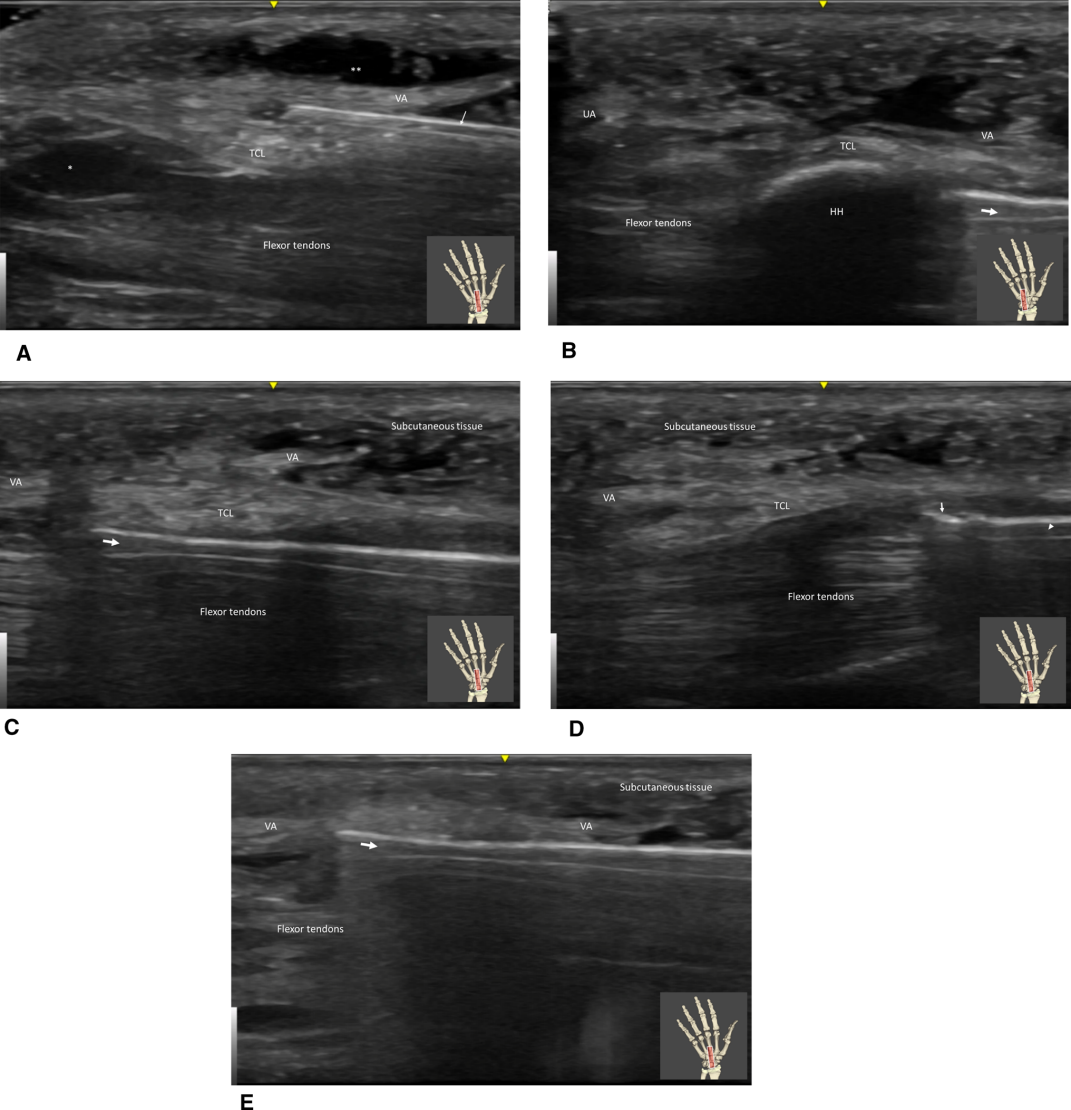
A Carpal tunnel in long axis view after xylocaïn injection under (*) the transverse carpal ligament (TCL) and over (**) the volar aponeurosis (VA). Contrast enhanced by local anesthesia injection improve the visualisation of the ligament. The puncture needle is locate between the TCL and the volar aponeurosis (arrow). B Carpal tunnel in long axis view passing at the level of the hamate hook (HH). The 1.5 mm probe (arrow) introduced through the puncture point is use to palpate anatomical structures like hamate hook (HH) to devine the best release trajectory. Note the ulnar artery (UA) crossing to become the superficial volar arch and the volar aponeurosis just above the transverse carpal ligament (TCL). C Carpal tunnel in long axis view passing just radial to the level of the hamate hook. The 1.5 mm probe (arrow) introduced through the puncture point is use to define the best release trajectory (along the safe zone). The 1.5 mm probe is moved from deep to superficial but remain in the carpal tunnel, stumbling over the transverse carpal ligament (TCL). Note the volar aponeurosis (VA) standing between the TCL and the subcutaneous tissue. D Carpal tunnel in long axis view passing just radial to the level of the hamate hook. The CT-SI Sonoinstrument (arrow head) is introduced through the puncture point and cut the transverse carpal ligament (TCL) along the safe zone. Note the cutting part of the instrument higlighted by the hyperechoïc line at its tip (arrow). Note the volar aponeurosis (VA) standing between the TCL and the subcutaneous tissue distally. E Carpal tunnel in long axis view passing just radial to the level of the hamate hook. After the release the 1.5 mm probe (arrow) introduced through the puncture point to palpate the potential ligament remnant. The 1.5 mm probe is moved from deep to superficial and is able to pass softly from the carpal tunnel, to the deep aspect of the volar aponeurosis (VA) or to the subcutaneous tissue if complete release is achieved

**Fig. 4:**
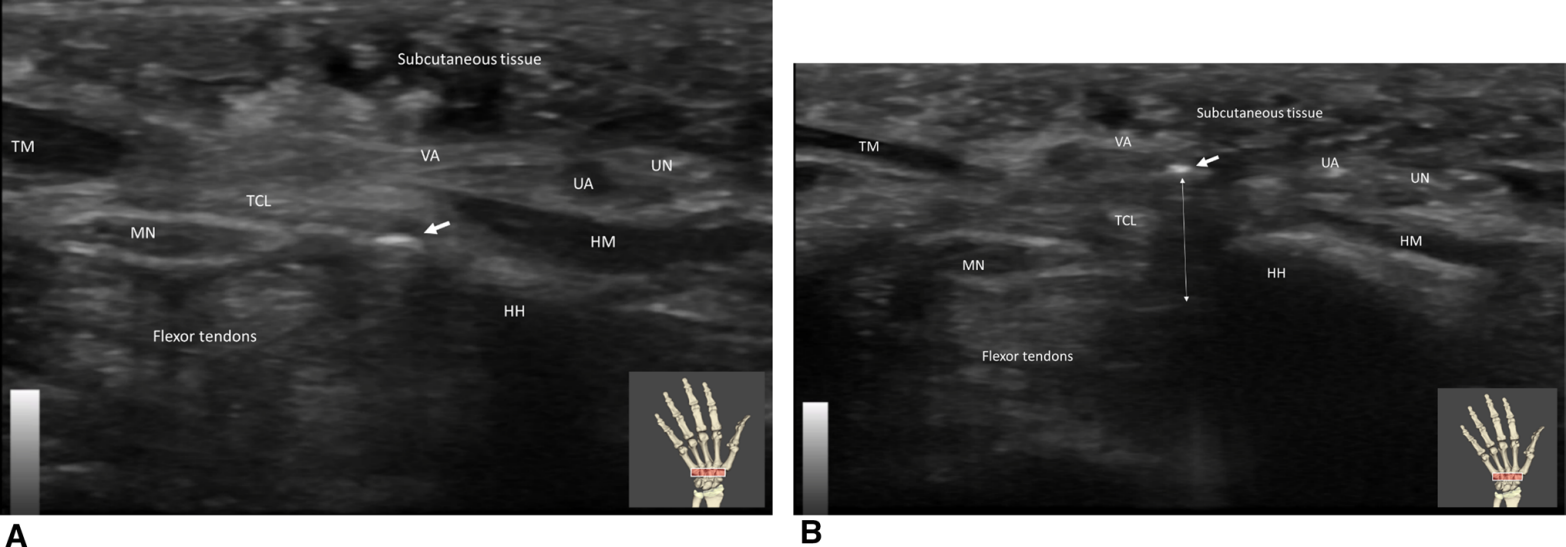
A Carpal tunnel in short axis view passing just at the level of the hamate hook (HH). The 1.5 mm probe (arrow) introduced through the puncture point is use to define the best release trajectory (along the safe zone: between the hamate hook and the median nerve (MN)). The 1.5 mm probe is moved from deep to superficial but remain in the carpal tunnel, stumbling over the transverse carpal ligament (TCL). Note the volar aponeurosis (VA) standing between the TCL and the subcutaneous tissue. *HM* Hypothenar muscle, *TM* Thenar muscle, *UA*Ulnar artery, *UN* Ulnar nerve. B Carpal tunnel in short axis view passing just radial to the level of the hamate hook (HH). After the release the 1.5 mm probe (arrow) introduced through the puncture point, the probe is moved from deep to superficial and is able to pass freely (double arrow) from the carpal tunnel to the deep aspect of the volar aponeurosis (VA) or to the subcutaneous tissue through the transverse carpal ligament (TCL)once complete release is achieved. Note the relative distance between the TCL defect and the median nerve (MN). *HM* Hypothenar muscle, *TM* Thenar muscle, *UA*Ulnar artery, *UN* Ulnar nerve

**Fig. 5:**
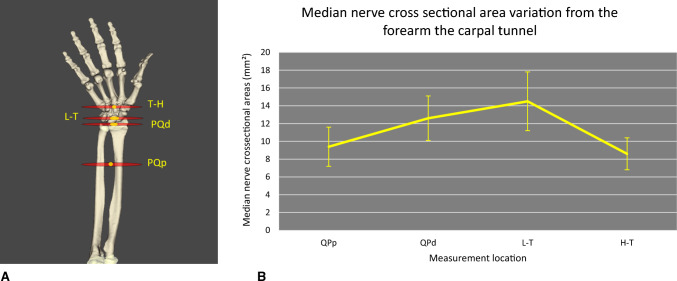
**A** Landmarks corresponding to the level of median nerve CSA measurements: proximal border of pronator quadratus muscle (QPp), distal border of the muscle (QPd), luno-triquetrum joint (L–T) and plane between hamate hook and trapezium tubercle (H-T). **B** measurements

The preoperative sonographic evaluation assessed for Lanz classification for the type of median nerve motor branch [26], Ferrari and Gilbert classification for the type of Berrettini anastomosis [27], and Lippert and Pabst classification for the type of superficial palmar arch [28]. The cross-sectional area (CSA) of the median nerve and the thickness of the TCL were measured at four locations defined by muscle or bony landmarks: proximal edge of the pronator quadratus, distal border of pronator quadratus, triquetro-lunate joint and in the plane joining the hook of hamate to the tubercle of the trapezium (Fig. 5A).

The limb was then disinfected and draped. The ultrasound probe was covered by a sterile plastic pouch, and sterile ultrasound gel was used. A 12-MHz linear sonography probe was used. Local anesthesia was performed under sonographic control, on the surface and under the TCL. A 14 Gauge catheter puncture through the skin and through the fascia allowed to introduce first a 1.5mm probe, to palpate the TCL, and to determine the safe zone of release. The SI was introduced, and progressive release was performed under sonographic control by progressive oscillating/cutting movements. Completion of the release was confirmed by re-introducing the probe and assessing its passage through the TCL to the superficial tissues. In case of any doubt concerning the completeness of the release, the SI was reinserted.

#### TF surgery: (Figs. 6, 7)

**Fig. 6:**
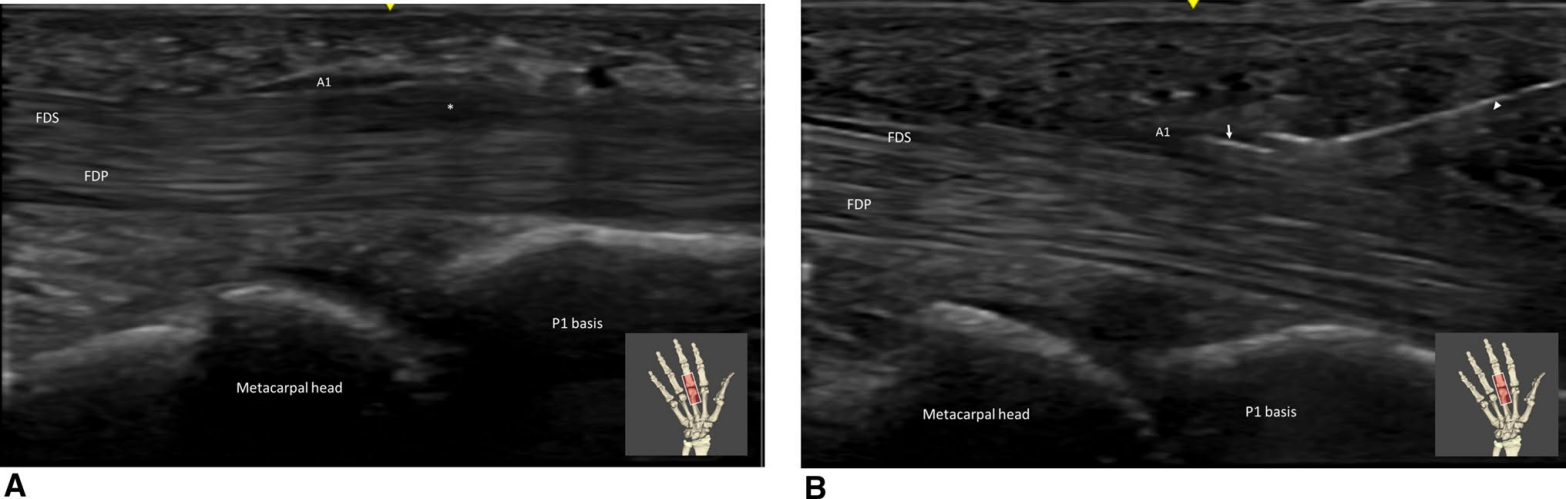
A Trigger finger (medius) in long axis view. The A1 pulley (A1) is thickened as well as the flexor digitorum superficialis tendon (FDS) with impingement between both structures. A1 pulley proximal and distal edges may correspond to the junction between the metacarpal neck and head and the junction between phalanx basis and diaphysis respectively. *FDP* Flexor digitorum profundus tendon. B Trigger finger (medius) in long axis view. A1 pulley is released with TF-Sonoinstrument (arrowhead). Note the cutting part of the instrument higlighted by the hyperechoïc line at its tip. *FDS* flexor digitorum superficialis tendon, *FDP* Flexor digitorum profundus tendon

**Fig. 7:**
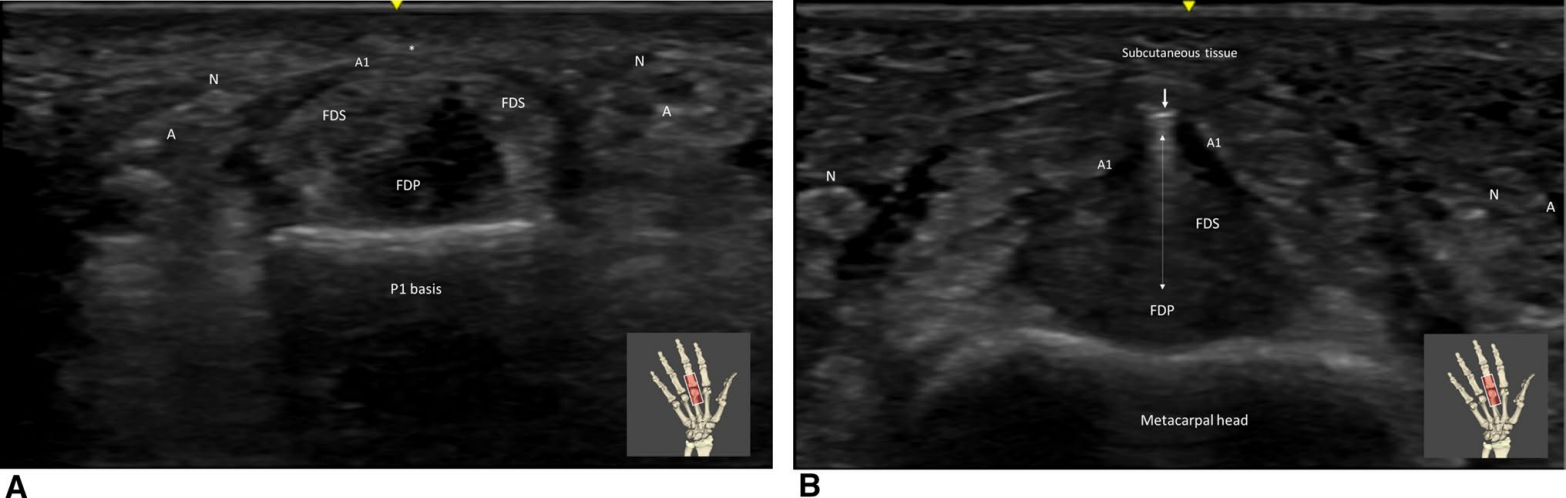
A Trigger finger (medius) in short axis view. At the level of the phalanx (P1) basis, before the release the A1 pulley (A1) is in closed contact with flexor tendons (FDS (Flexor digitorum superficialis) and FDP (Flexor digitorum profundus). Note the location of the radial and ulnaire digital neurovascular bundles (A (artery) and N (nerve)) quite far from the A1Pulley apex (*). 7B: Trigger finger (medius) in short axis view. At the level of the metacarpal head, after the release the percutaneaous A1 pulley (A1) release. The TF-SI Sononinstrument (arrow) is passing freely (double arrow) between the FDS (Flexor digitorum superficialis) and FDP (Flexor digitorum profundus) digital sheath and the subcutaneous tissue. Note the location of the radial and ulnar digital neurovascular bundles (A (artery) and N (nerve)) quite far from the released area

The preoperative sonographic evaluation involved the assessment of tendon gliding under the A0/A1 pulley, state of the flexor tendons, and A1 pulleys thickness. A hockey-stick 18MHz sonography probe was used. Local anesthesia was performed under sonographic control, on the surface and under A1 pulley. The anesthesia aided in observing the triggering phenomenon if the finger was too painful to mobilize. A 14 Gauge catheter puncture through the skin and tendon sheath allowed to introduce the SI (the probe was most of the times not used). Progressive release was obtained under sonographic control through progressive oscillating/cutting movements. Completion of release was assessed through observation of the absence of catching whilst actively moving the finger and through sonographic evaluation of the improved gliding of the flexor tendons. In case of any doubt concerning the completeness of the release, the SI was reinserted.

### Post operative protocol

A compressive dressing was applied, which could be removed after a few hours. The patients were instructed to immediately start active mobilization of the fingers. In case of persistent lack of extension in the PIP joint, passive stretching of the joint was advised. In case of CTS, patients were instructed (1) to wear a sling for few days, (2) to elevate the operated limb, (3) to move the fingers actively to prevent swelling and complex regional pain syndrome, and (4) to avoid for several weeks heavy work with the wrist in palmar flexion.

### Follow-up

The patients were followed-up for two months, with programmed visits at one week and at two months. In case the patient was unable to come to the clinic, data was obtained by telephonic interview.

### Statistics analysis

Continuous variables were expressed as mean (SD), median, range, and 95% CI, while categorical variables were shown as number, denominator, percentage, and 95% Clopper Pearson interval. Two-sided statistical tests were performed at a 5% significance level, with changes from baseline tested via paired Student’s *t* test (continuous variables) or Wilcoxon Signed Rank test (categorical variables). Tests with *p*-values < 0.05 were statistically significant. Missing data were reported but not imputed.

## Results

In the CTS group, the average age of the 15 patients was 58.7 ± 15.1 years, with 12 women (80%) and 3 men (20%). Of the patients, 26.7% (4/15) were active smokers and 13.3% (2/15) were diabetics. The patients had symptoms for 20.7 ± 10.0 months before the operation, and their average DASH was 50.6. Diagnosis of CTS was confirmed by pre-operative EMG with an average distal motor latency of 4.9 ± 1.7 ms.

The pre-operative sonographic findings are reported in Table 2. The mean CSA of the median nerve increased from the forearm to the begin of the carpal tunnel, and suddenly decreased in the carpal tunnel and the differences were highly significant (*p* < 0.001). Specifically, the CSA at proximal edge of the pronator quadratus measured 9.4 ± 2.2 mm^2^, and at distal edge it was significantly larger at 12.6 ± 2.5 mm^2^ (*p* = 0.001). The CSA further enlarged at the luno–triquetral joint (14.5 ± 3.3 mm^2^; *p* < 0.001 when compared to the proximal edge), and then decreased to 8.6 ± 1.8 mm^2^ (Fig. 4B). The percutaneous section of the TCL was always successful, with excellent device performance and no adverse events (Table 3). The mean operative time was 10.5 min (SD ± 3.0).

**Table 2:** Pre-operative ultrasound findings

CT group 15 patients		TF group 15 patients	
Type of median nerve motor branch: Lanz classification [26]		Tendon sliding under A0/A1 pulley	100.00% (15/15)
0 – Extra-ligamentous thenar branch	13.3% (2/15)	Abnormal sliding FDS	6.7% (1/15)
1A—Subligamentous thenar branch	73.3% (11/15)	FDS blockade	6.7% (1/15)
1B—Transligamentous thenar branch	6.7% (1/15)	FDS and FDP blockade	40% (6/15)
4A—Proximal accessory thenar branch running directly in the thenar muscles	6.7% (1/15)	FPL altered gliding	26.7% (4/15)
Type of Berrettini anastomosis: Ferrari and Gilbert Classification[27]		Superficial catcher	6.7% (1/15)
Type 3	100% (15/15)	Thickening of FDS	60% (9/15)
Type of superficial palmar arch: Lippert and Pabst (LP) classification)[28]		Radial slip	11.1% (1/9)
Complete (LP-A)	33.3% (5/15)	Ulnar slip	77.8% (7/9)
LP-A1	60% (3/5)	A1 pulley parameters	
LP-A4	40% (2/5)	Length (cm)	1.0 ± 0.3
Incomplete (LP-B)	66.7% (10/15)	Thickness (mm)	1.2 ± 0.4
LP-B1a	30% (3/10)		
LP-B1b	40% (4/10)		
LP-B2	20% (2/10)		
LP-AB	10% (1/10)		
Thickness of TCL (mm)	1.5 ± 0.3		
Other Abnormalities			
Presence of superficial radial artery	40.0% (6/15)		
Generalized muscle atrophy	100.0% (1/1)		
Hypertrophic hook of hamatum	13.3% (2/15)		
Atrophy of thenar muscles	20.0% (3/15)		
Atrophy of lumbrical muscles	20.0% (3/15)		

*FDS* flexor digitorum superficialis. *FDP* flexor digitorum profundus

**Table 3:** Device performance assessment

	CTS group	TF group
Device performance assessment (each item score/4):		
Ergonomic design	4.0 ± 0.0	4.0 ± 0.0
Visibility of marking on the handle	4.0 ± 0.0	4.0 ± 0.0
Usability of marking on the handle	1.8 ± 0.7	1.6 ± 0.5
Visibility of marking under sonography	1.7 ± 0.4	2.6 ± 0.4
Usability of marking under sonography	1.3 ± 0.4	1.2 ± 0.4
Visibility of cutting extremity under sonography	2.9 ± 0.2	3.6 ± 0.4
Ease of positioning, maneuverability	3.9 ± 0.2	4.0 ± 0.0
Release of TCL at carpal tunnel	3.8 ± 0.4	N/A
Release of carpi volare	3.2 ± 0.5	N/A
Release of A0/A1 pulley	N/A	3.8 ± 0.3
Device stability	4.00 ± 0.0	4.0 ± 0.0
Device maneuverability	3.9 ± 0.2	4.0 ± 0.0
Device consistency and reliability	3.8 ± 0.3	4.0 ± 0.0
Total device performance score (score/48 for CTS, score/44 for TF)	38.4 ± 1.6	37.0 ± 1.6
Probe performance (score/12)	12.0 ± 0.0	8.0 ± 0.0
Technical success and efficiency of performing the intervention	100.00% (15/15)	100.0% (15/15)
Operating time (minutes), mean ± SD	10.47 ± 2.97 (15)	7.93 ± 3.67 (15)
Bleeding during procedure		
No bleeding	93.3% (14/15)	100,0% (15/15)
Venous bleeding	6.6% (1/15)	0% (0/15)
Arterial bleeding	0% (0/15)	0% (0/15)

*CTS* Carpal tunnel syndrome. *TF* Trigger finger. SD: Standard deviation

At one week, all patients could wash their hands, 80% could perform all activities of daily living, and 80% of those working had returned to their professional activities. However, 40% still complained of pain and/or numbness at night. The mean DASH was 44.7 ± 20.0 with other functional scores reported in Table 4. At two months, all patients had resumed all activities of daily living and work, and only 20% complained of mild persistent pain and/or numbness at night. The mean DASH was 29.4, with pillar pain reported by 53.3% of the patients (Table 4).

**Table 4:** Results of carpal tunnel syndrome series

Clinical effectiveness assessment	Baseline	CT	
		Week 1	Month 2
NRS (in rest and activity)			
Rest (average)	*3.3* ± *2.7*	2.2 ± 2.6 (*p* = *0.264)*	1.6 ± 2.1 (*p* = *0.030)*
Activity (average)	*5.0* ± *1.9*	3.6 ± 2.5 (*p* = *0.143)*	3.2 ± 2.7 (*p* = *0.028)*
Use of medication			
Painkillers	*40.0% (6/15)*	66.6% (10/15) (*p* = *0.218)*	13.3% (2/15) (*p* = *0.125)*
NSAID	*33.3% (5/15)*	26.6% (4/15) (*p* = *1.000)*	6.6% (1/15) (*p* = *0.125)*
Total Quick DASH [24]	*50.6* ± *22.7*	44.7 ± 19.9 (*p* = *0.322)*	29.3 ± 24.0 (*p* = *0.001)*
Total BwCTQ-SS [25]	*2.9* ± *0.8*	2.0 ± 0.7 (*p* = *0.002)*	1.7 ± 0.8 (*p* = *0.000)*
Total BCTQ-FS [25]	*2.5* ± *0.8*	2.3 ± 0.8 (*p* = *0.589)*	1.9 ± 0.9 (*p* = *0.023)*
Numbness/pain at night	*100.0% (15/15)*	40.0% (6/15) (*p* = *0.003)*	20.0% (3/15) (*p* = *0.0005)*
Pillar pain		86.6% (13/15)	53.3% (8/15)
Hand washing		100.0% (15/15)	100.0% (15/15)
Return to activities of daily living		80.0% (12/15)	100.0% (15/15)
Effective time to return to activities of daily living (days)		6.2 ± 5.3 (N = 15)	
Return to work		80.0% (4/5)	100.0% (5/5)
Effective time to return to work (days)		10.4 ± 7.0 (5)	

*NRS* Numeric Pain Rating Scale. *DASH* Disabilities of the Arm, Shoulder, and Hand. *BCTQ-SS* Boston Carpal Tunnel Questionnaire—Symptom Severity Scale. *BCTQ-FS* Boston Carpal Tunnel Questionnaire—Functional Status Scale. *NSAID* Nonsteroidal Anti-inflammatory Drug

In the TF group, the average age of the 15 patients was 57.9 ± 8.0 years. There were 7 women (46.7%) and 8 men (53.3%). Four patients (26.7%) were active smokers and 1 (6.7%) was diabetic. There were four thumbs and 11 digits operated on. The patients had been suffering on average 8.1 ± 5.3 months of triggering symptoms before the operation, and their average DASH was 45.3.

The average thickness of the A1 annular pulley was 1.2 ± 0.4 mm. We observed abnormal gliding of the tendons in 100% (15/15), and, in transverse view, thickening of the FDS ulnar slip in 77.8% and of the radial slip in 11.1% (Table 2). The percutaneous section of the A1 annular pulley was always successful, with excellent device performance and no adverse events (Table 3). The mean operative time was 7.9 min (SD ± 3.7min).

At one week, all patients could wash their hands, 93.3% could do all activities of daily living, and 75% of those working were back to their professional activities. Most patients had already recovered full motion (93.3%). At two months, all patients were back to all activities of daily living and of work, and all patients had recovered normal motion. The mean DASH was 17.7 ± 18.3 (Table 5). One patient complained of persistent pain at the level of the A0 pulley, without triggering. He was re-operated for further release at this level at two months.

**Table 5:** Results of trigger finger series

Clinical effectiveness assessment (Baseline versus follow-up)	Baseline	TF	
		Week 1	Months 2
NRS (in rest and activity)			
Rest (average)	2.9 ± 2.6	1.0 ± 1.2 (*p* = *0.010)*	1.2 ± 2.3 (*p* = *0.048)*
Activity (average)	5.3 ± 2.3	2.8 ± 1.6 (*p* = *0.005)*	2.0 ± 2.4 (*p* = *0.0006)*
Use of medication			
Painkillers	26.6% (4/15)	40.0% (6/15) (*p* = *0.687)*	0.0% (0/15) (*p* = *0.125)*
NSAID	13.3% (2/15)	0.0% (0/15) (*p* = *0.500)*	0.0% (0/15) (*p* = *0.500)*
Total Quick DASH [24]	45.3 ± 19.6	23.3 ± 16.0 (*p* = *0.000)*	17.7 ± 18.2 (*p* = *0.0003)*
Quinnel grading [22]			
Normal movement	0% (0/15)	93.3% (14/15) (*p* < *.0001)*	100.0% (15/15) (*p* < *.0001)*
Uneven movement	40.0% (6/15)	6.6% (1/15)	0% (0/15)
Actively correctable	40.0% (6/15)	0% (0/15)	0% (0/15)
Passively correctable	13.3% (2/15)	0% (0/15)	0% (0/15)
Fixed deformity	6.6% (1/15)	0% (0/15)	0% (0/15)
Modified Patel and Bassini’s grading system [23]			
Improvement in pain			
Complete resolution		20.0% (3/15)	86.6% (13/15)
Good improvement		60.0% (9/15)	6.6% (1/15)
Some improvement		13.3% (2/15)	6.6% (1/15)
No improvement		6.6% (1/15)	0.0% (0/15)
Improvement in triggering			
Complete resolution		93.3% (14/15)	100.0% (15/15)
Good improvement		0.0% (0/15)	0.0% (0/15)
Some improvement		6.6% (1/15)	0.0% (0/15)
No improvement		0.0% (0/15)	0.0% (0/15)
Hand washing		100% (15/15)	100% (15/15)
Return to activities of daily living		93.3% (14/15)	100% (15/15)
Effective time to return to activities of daily living (days)		2.8 ± 2.4 (15)	
Return to work		75,0% (3/4)	100% (15/15)
Effective time to return to work (days)		3.5 ± 4.3 (4)	

*NRS* Numeric Pain Rating Scale. *NSAID* Nonsteroidal Anti-inflammatory Drug. *DASH* Disabilities of the Arm, Shoulder, and Hand

## Discussion

This study was undertaken to evaluate the safety and effectiveness of the SI, a novel medical device allowing truly percutaneous treatment of CTS and TF through a single needle puncture. No safety concerns were observed during the course of this study. All operations were completed efficiently under local anesthesia. All patients showed significant improvement after the operation. No complications related to the use of the SI were observed. One patient presented persistent pain after TF release, due to a conflict of the tendons under the A0 pulley which was not released at the time of index surgery.

The SI allows a safe release of the ligament/pulley by permitting an optimal visualization of all important anatomical structures, including dangerous situations as Lanz group 1 thenar branch [13, 26], Ferrari type 1 Berrettini branch in CTS [27, 29], or a distally crossing thumb radial digital nerve in trigger thumb [30]. The risk of iatrogenic injury is further reduced by the design of the cutting extremity of the SI, which is effective on tense ligament fibers, but not acerate enough to injure inadvertently tendons, vessels, or nerves. Medical devices comprising a sharp cutting blade might expose to a higher risk of iatrogenic injuries, especially when the patient has anatomical variations or simply when the cutting procedure goes beyond needed, due to forceful manipulations [17–20]. Furthermore, some of these other devices are not adapted to thick TCL or hypertrophied annular pulleys. The sole advantage of them, when compared to SI, is that, when in place after open dissection, they allow quicker release, which is a difference of a few minutes [17, 18]. But this will necessitate a step of surgical dissection, not needed with SI.

**Fig. 8:**
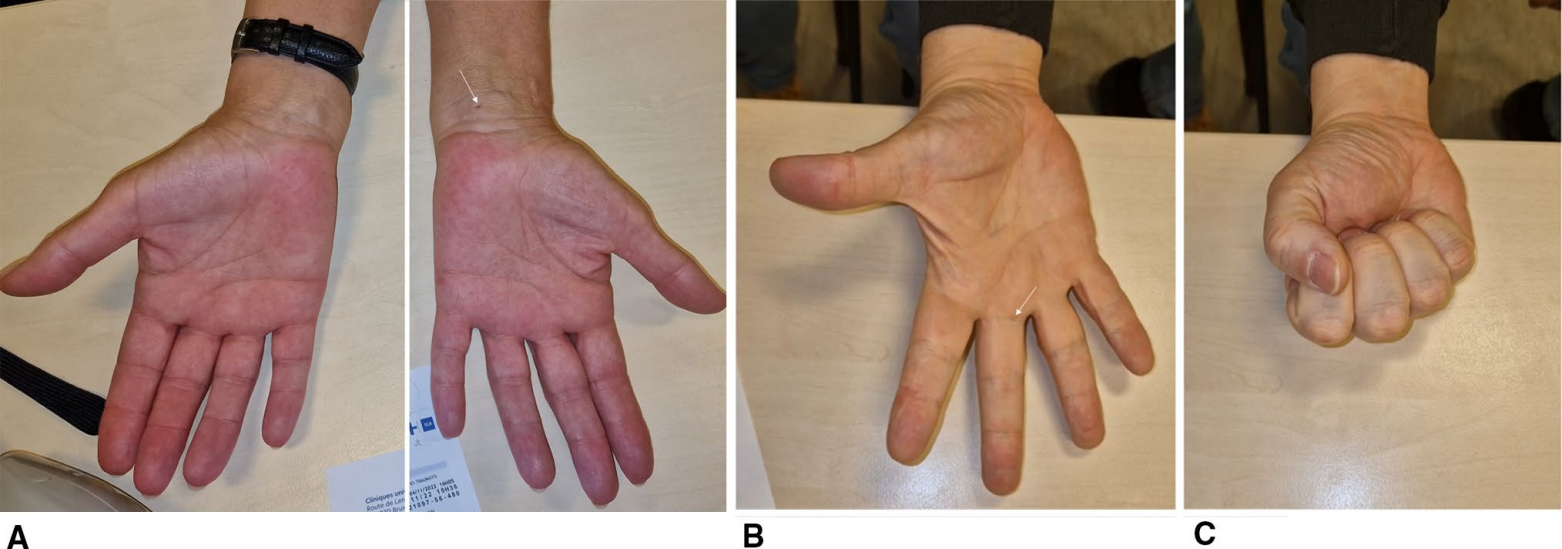
Seven days after percutaneous sonoguided treatment using SI. A CTS treatment, left side. The entry point scar is the only visible sign of the operation (arrow). **B,C** TF operation, middle finger. Note the minimal scar of entry point (arrow). The patient is able to fully close the hand fist, without pain nor limitation

SI allows truly percutaneous surgery. As there is no skin incision, there are no stitches nor postoperative dressings. Patients can wash their hands the next day, and immediately resume their daily activities (Fig. 8). Due to the absence of any wounds in the palm, patients experience minimal pain when using their hand for various activities during the initial postoperative days. Using another device, Eberlin et al. also reported that percutaneous procedure is associated with decreased direct wound pain [19].

Though the present study was performed in the operating room of an ambulatory facility for legal reasons (pre-CE mark clinical investigation), percutaneous release of CTS and TF using SI seems to be particularly well adapted to office surgery, markedly diminishing the costs of the operation and the stress experienced by the patient [31]. Indeed, this study demonstrates the safety of the procedure. No surgical instrument is needed except the SI and the probe, conveniently disposed in the single-use sterile kit provided by the manufacturer. No tourniquet is needed, on the contrary a tourniquet should be avoided as it prevents the visibility of pulsating arteries under sonography. The operation is done under local anesthesia, either standard or WALANT [32], and no significant bleeding is to be expected. The operation takes only a few minutes. Bilateral surgery is possible, just like simultaneous release of several TFs or concomitant CTS and TF surgery (such cases were excluded in our protocol, to avoid any confusion in the interpretation of the results). These potential advantages for office surgery need to be confirmed in future prospective studies.

Whilst in this study, we could not demonstrate an accelerated return to working activities, in the absence of a control group, previous studies have reported faster return to work after minimally invasive procedures such as ultrasound-guided and endoscopic carpal tunnel release procedures [5, 33]. Rojo-Manaute et al. conducted a randomized clinical trial to compare ultrasound-guided CTS and open CTS and found a significantly faster return to daily activities, as early as 4.9 days, as compared to 25.4 days for open carpal tunnel release[34]. Asserson et al. retrospectively compared two groups of similar techniques and found a rapid return to work, as early as 12 days, for ultrasound-guided operations, compared to 33 days after open surgery [34, 35].

Percutaneous treatment of CTS does not avoid pillar pain, as seen in all operative methods of treating the condition. Pillar pain is probably related to the healing of the sectioned TCL [36, 37]. The patients should be informed before the operation of this foreseeable complication. Especially, as the patients have no wounds or scars after percutaneous surgery (Fig. 8), they believe the operation is similar to an injection and do not expect to experience this painful condition after percutaneous surgery.

Our study is limited by the small sample size of the groups and the absence of a control group. However, the results of this study provide evidence for the effectiveness and safety of percutaneous treatment of CTS and TF using SI.

Percutaneous release using SI is a new form of surgery that needs training and experience. Henceforth, surgeons need to master hand sonography, to dispose of a high-frequency sonograph in the operation room, and to learn how to easily manipulate the SI in the volume of the probe, constantly seeing well the instrument, and the technique of progressive cut by small repeated movements. Training on a mannequin hand and/or a cadaveric specimen is mandatory before the first cases, and it is probably reasonable for the surgeons to start with digits TF cases, before operating TF thumb and CTS.

In this study, we prospectively evaluated the safety and effectiveness of a new minimally invasive device, the Sono-Instrument®, for the treatment of CTS and TF. The main findings of this study are that SI are safe and efficient and constitute an attractive alternative to traditional open surgical or endoscopic techniques and to other ultrasound-guided techniques. The device seems to be particularly adapted to office surgery.

## Data Availability

Data are available on reasonable request.
